# Advances in cold-adapted enzymes derived from microorganisms

**DOI:** 10.3389/fmicb.2023.1152847

**Published:** 2023-04-26

**Authors:** Yehui Liu, Na Zhang, Jie Ma, Yuqi Zhou, Qiang Wei, Chunjie Tian, Yi Fang, Rongzhen Zhong, Guang Chen, Sitong Zhang

**Affiliations:** ^1^College of Life Science, Jilin Agricultural University, Changchun, China; ^2^Key Laboratory of Straw Comprehensive Utilization and Black Soil Conservation, Ministry of Education, Changchun, China; ^3^Key Laboratory of Mollisols Agroecology, Northeast Institute of Geography and Agroecology, Chinese Academy of Sciences, Changchun, China

**Keywords:** cold-adapted enzymes, psychrophile, cold-adapted catalytic mechanism, molecular modification, application of cold-adapted enzymes

## Abstract

Cold-adapted enzymes, produced in cold-adapted organisms, are a class of enzyme with catalytic activity at low temperatures, high temperature sensitivity, and the ability to adapt to cold stimulation. These enzymes are largely derived from animals, plants, and microorganisms in polar areas, mountains, and the deep sea. With the rapid development of modern biotechnology, cold-adapted enzymes have been implemented in human and other animal food production, the protection and restoration of environments, and fundamental biological research, among other areas. Cold-adapted enzymes derived from microorganisms have attracted much attention because of their short production cycles, high yield, and simple separation and purification, compared with cold-adapted enzymes derived from plants and animals. In this review we discuss various types of cold-adapted enzyme from cold-adapted microorganisms, along with associated applications, catalytic mechanisms, and molecular modification methods, to establish foundation for the theoretical research and application of cold-adapted enzymes.

## 1. Introduction

Cold-adapted enzymes, also called psychrophilic enzymes, are enzymes that can effectively catalyze low temperature reactions and are very temperature sensitive (Kulakova et al., 1999). As defined by Margesin and Schinner (1992) enzymes that optimally catalyze at about 30°C and still have some catalytic efficiency at 0°C are usually called cold-adapted enzymes. Unlike mesophilic and thermophilic enzymes, cold-adapted enzymes have the following characteristics: (1) The optimal reaction temperature is low, with an optimal catalytic temperature generally between 20°C to 45°C. (2) The enzymatic reaction activation energy is low and can be reduced by increasing substrate turnover or affinity. (3) Thermal stability is low, with rapid, easily lost activity at high temperatures (more than half of activity is lost after 10 min at 50°C to 60°C or several hours at 37°C) (Feller et al., 1997; Berchet et al., 2000; D'Amico et al., 2003; Santiago et al., 2016).

Baldwin and Hochachka (1970) discovered the existence of a cold-adapted enzyme in trout brains and began theoretical and applied research on it. The first paper to biochemically characterize cold-adapted enzymes was published in 1984, in which the optimal temperature and substrate preferences of a thermosensitive alkaline phosphatase from an Antarctic bacterium were analyzed in detail (Kobori et al., 1984). Through continued biotechnological development, cold-adapted enzymes have been applied in food processing, detergent production, basic molecular biology research, and other areas.

Cold-adapted enzymes, because their loose structure and high flexibility, reduce the activation energy required for enzymatic reactions at lower temperatures. But this also results in the disadvantage of increased sensitivities to metallic ions and organic solvents, and a low variability of thermal stability. Furthermore, a lack of enzymatic sources and difficulty in separating and purifying these enzymes seriously limit the industrial application of cold-adapted enzymes. Regardless, compared with cold-adapted enzymes from animals and plants, cold-adapted enzymes from microorganisms have the positive characteristics of abundant source, short production cycle, high yield, and simple separation and purification, and thus have attracted much attention (Georlette et al., 2000; Kim et al., 2018; Ge et al., 2020).

Cold-adapted enzymes have been observed in *Pseudomonas*, *Penicillium*, and the yeast *Rhodotorula*, among many microorganisms. Investigations into cold-adapted catalytic mechanisms and the molecular modifications of cold-adapted enzymes have frequently been reported (Kumar et al., 2021). However, research and reports on applications of cold-adapted enzymes from microorganisms are dispersed and unsystematic to date. This review systematically discusses the types, applications, catalytic mechanisms, and molecular modifications of cold-adapted enzymes from microorganisms, providing a theoretical basis for the research and comprehensive application of cold-adapted enzymes.

## 2. Principal types of cold-adapted enzymes from microorganisms

Approximately 100 types of cold-adapted enzymes have been reported over the last few years (Santiago et al., 2016; Pereira et al., 2017; DangThu et al., 2020). According to International Commission of Enzymes nomenclature, these enzymes can be classified into six primary categories: hydrolase, oxidoreductases, ransferases, lyases, isomerases, and synthetases. Because there are six types of cold-adapted enzymes involved, we selected typical cold-adapted enzymes for introduction.

### 2.1. α-Amylases from cold-adapted hydrolases

Hydrolases are the most common commercially used cold-adapted enzyme of the six primary categories. These include cold-adapted proteases, lipases, and amylases. Among them, α-amylases was the first cold-adapted hydrolase to be crystallized and analyzed. *Bacillus, Cryptococcus*, *Penicillium*, *Pseudomonas*, and the yeast *Rhodotorula* are principal representative genera used for the production of cold-adapted α-amylases. Commercial cold-adapted microbial α-amylases and associated characteristics are overviewed in Table 1.

**Table 1:** Cold-adapted α-amylases from various microorganisms and associated characteristics.

Enzyme	Microorganism	Optimal temperature	Optimal pH	Deactivation conditions	Activator	Inhibitors	Reference
α-Amylase	*Arthrobacter agilis* PAMC 27388	30°C	3.0	50°C, 10 min	Co^2+^, Fe^3+^, Na^+^, K^+^, β-ME	Mg^2+^, Zn^2+^, urea, SDS, EDTA	Kim et al. (2017)
Amy175	*Pseudoalteromonas* sp. M175	25°C	8.0	55°C, 10 min	Ca^2+^, Mg^2+^, Zn^2+^, K^+^, EDTA, SDS, Triton X-100	Co^2+^, β-ME	Wang Y. et al. (2018); Wang X. et al. (2018)
Amy SH3	*Exiguobacterium.*	37°C	7.0	50°C, 20 min	Co^2+^	Ca^2+^, Mg^2+^, Urea, Zn^2+^, Fe^3+^, SDS, β-ME, EDTA	Mojallali et al. (2014)
Amylase	*Nocardiopsis* sp. 7,326	35°C	8.0	60°C, 10 min	Co^2+^, Ca^2+^, K^+^, Mg^2+^, Zn^2+^	EDTA	Zhang and Zeng (2008)
Amylase GS230	*Pseudoalteromonas arctica* GS230	30°C	7.5	50°C, 10 min	Co^2+^, Urea, Ca^2+^, Mg^2+^, Na^+^, K^+^	Fe^3+^, Zn^2+^, EDTA, SDS	Lu et al. (2010)

**Figure 1:**
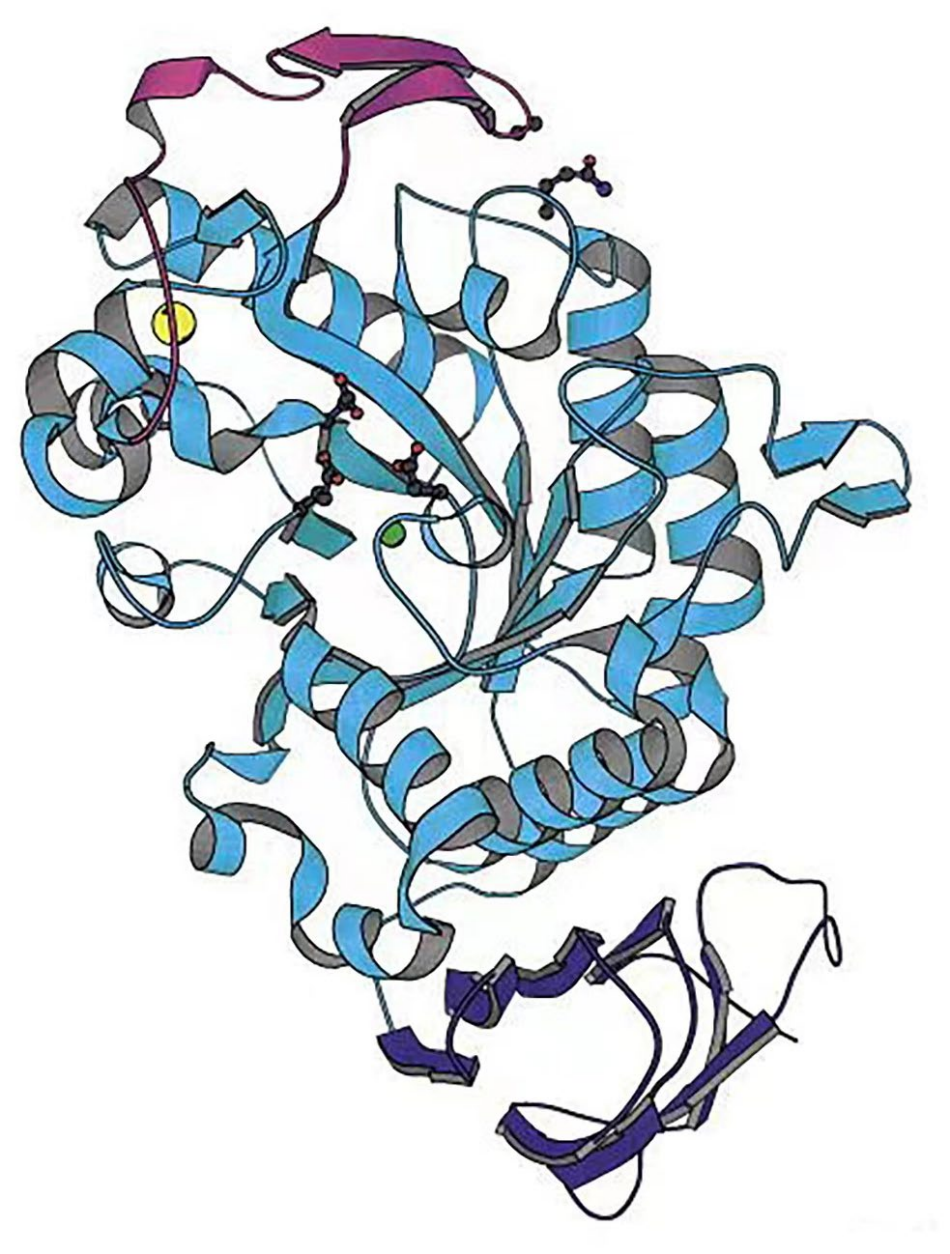
Cold-adapted α-amylase from *Alteromonas haloplantis* structure (Aghajari et al., 1998).

Aghajari et al. (1998) analyzed the structure of an *Alteromonas haloplanctis* cold-adapted α-amylase (AHA) and explained its cold catalytic mechanism at a molecular level by comparison with the structure of human pancreatic α-amylase (HPA) (Figure 1). AHA is a typical monomeric enzyme, with three domains A, B, and C. Unlike HPA, AHA has a large number of amino acid residues that are eliminated. The number of hydrogen bonds, glutamate residues, aspartate residues, and proline residues in domains A consisting of a (β/α) _8_-barrel structures, and in domain C consisting of eight β-strands, and in its N-terminal domain, are significantly lower than so in HPA. The number of disulfide bonds in the B domain is also significantly lower. This makes AHA’s structure looser than HPA’s, increasing AHA’s affinity for substrates (Aghajari et al., 1998). Unlike mesophilic and thermophilic α-amylases, most cold-adapted α-amylases are highly active at 25–37°C and pH 4.0–8.0, are inactivated at 50°C for 10–15 min, and are sensitive to metal and salt ions, and organic solvents (Lu et al., 2010; Kim et al., 2017). However, cold-adapted enzyme resource development has found some cold-adapted α-amylases that possess a degree of tolerance to metal and salt ions. For instance, Wang Y. et al. (2018) and Wang X. et al. (2018) analyzed and characterized a cold-adapted α-amylase from an Antarctic bacterium and found the enzyme to be extremely salt-tolerant. It also possesses a degree of tolerance to other metal ions, such as potassium, calcium, and manganese, and to solvents, including sodium dodecyl sulfate (SDS), and dimethyl sulfoxide (DMSO). Therefore, this cold-adapted α-amylase is more suitable for use in detergents than most other α-amylases (Wang Y. et al., 2018; Wang X. et al., 2018).

### 2.2. Superoxide dismutase from cold-adapted oxidoreductase

To date, cold-adapted oxidoreductases derived from microorganisms mainly consist of sorbitol dehydrogenase, glutathione reductase, hydrogen peroxide reductase, and superoxide dismutase. Of these, cold-adapted superoxide dismutase is commonly used in clinical debridement and for other therapeutic purposes (DangThu et al., 2020; Wang Z. P. et al., 2020; Wang Q. et al., 2020; Krumova et al., 2021). Presently, *Pseudoalteromonas*, *Salmonella*, and the fungi *Aspergillus* are the primary sources of cold-adapted superoxide dismutases. Most show high activity at 25°C–35°C and pH 7.0–8.0 (Abrashev et al., 2016; Wang Z. P. et al., 2020; Wang Q. et al., 2020). Major features of microbial cold-adapted superoxide dismutases are summarized in Table 2.

**Table 2:** Cold-adapted superoxide dismutases from various microorganisms and associated characteristics.

Enzyme	Microorganism	Optimal temperature	Optimal pH	Deactivation conditions	Activator	Inhibitors	Reference
rHsSOD	*Halomonas* sp. ANT108	35°C	7.5	60°C, 20 min	Cu^2+^, Zn^2+^	Ca^2+^, Fe^3+^, Cd^2+^, Co^2+^, Cr^3+^, Ni^2+^, Mg^2+^, DTT, TritonX-100	Wang Z. P. et al. (2020); Wang Q et al. (2020)
PsSOD	*Pseudoalteromonas* sp. ANT506	30°C	8.0	70°C, 20 min	Ca^2+^, Mg^2+^, Cu^2+^, Zn^2+^, Tween-80	Ni^2+^, Fe^3+^, SDS, Urea	Wang et al. (2016)
PhSOD	*Pseudoalteromonas haloplanktis*	25°C	6.9	54°C, 10 min	Na^+^, EDTA	K^+^	Castellano et al. (2006)

**Figure 2:**
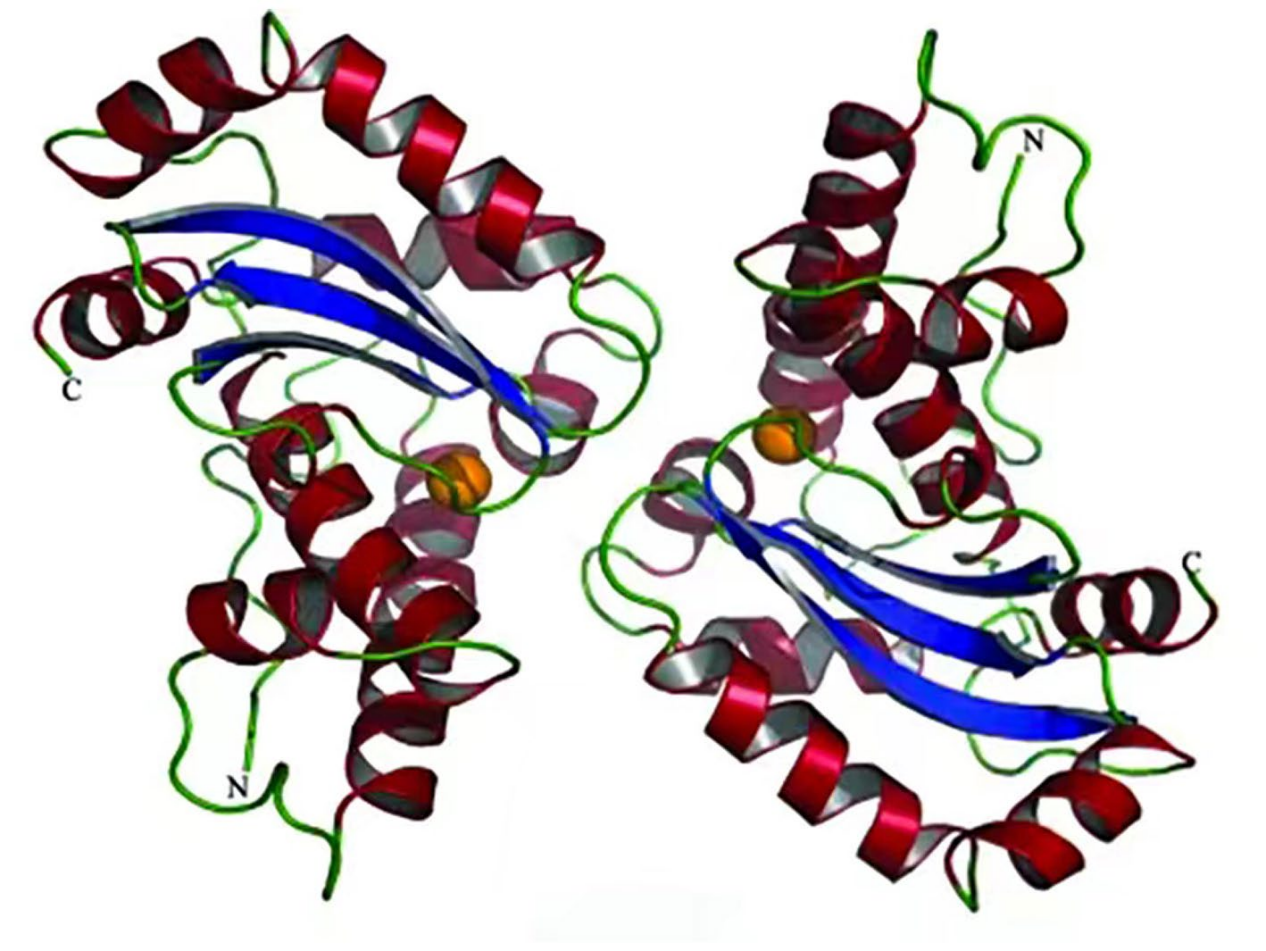
Superoxide dismutase from *Aliivibrio salmonicida* structure (Pedersen et al., 2009).

Pedersen et al. (2009) reported the structure of cold-adapted iron superoxide dismutase from *Aliivibrio salmonicida* analyzing its active site and pocket area (Figure 2), and found that the number of disulfide and hydrogen bonds in the region were significantly lower than that of homologous mesophilic iron superoxide dismutases. They attribute this decrease in the number of disulfide and hydrogen bonds to be the main factor in maintaining the flexibility of its structure (Pedersen et al., 2009). Interestingly, in contrast to other types of cold-adapted enzymes, the cold-adapted superoxide dismutase from *Halomonas* sp. ANT108 (rHsSOD) is itself a metalloenzyme, so that it is tolerant to Cu^2+^ and other metal ions. Therefore, Cu^2+^ and Zn^2+^ ions can promote rHsSOD activity in this bacterium, thus avoiding breaks in DNA strands caused by metal-catalyzed oxidation (Wang Z. P. et al., 2020; Wang Q. et al., 2020).

### 2.3. Alginate lyase from cold-adapted lyase

Cold-adapted lyases from microbes predominantly include glycosaminoglycan lyase, alginate lyase, and DNA photolyase. Among these, cold-adapted alginate lyases can cleave alginate to produce trehalase, which is an enzyme of high value with broad applications in industrial production processes (Munshi et al., 2017; Gao et al., 2018; Zhang et al., 2022). Researchers have studied cold-adapted alginate lyases from *Vibrio*, *Flavobacterium*, *Pseudoalteromonas* spp., and *Alteromonas portus*, finding that optimal catalytic conditions are generally between 25°C and 35°C and pH 7.0–8.0 (Chen et al., 2016; Wang Z. P. et al., 2020; Wang Q. et al., 2020; Zhou et al., 2020; Huang et al., 2021). Table 3 summarizes connercial cold-adapted alginate lyases of microbial origin and associated characteristics.

**Table 3:** Cold-adapted alginate lyases from various microorganisms and associated characteristics.

Enzyme	Microorganism	Optimal temperature	Optimal pH	Deactivation conditions	Activator	Inhibitors	Reference
ALG2951	*Alteromonas portus*	25°C	8.0	60°C, 20 min	Na^+^, K^+^, Na^+^	Mn^2+^, Fe^2+^, Ba^2+^, SDS	Huang et al. (2021)
AlyS02	*Flavobacterium* sp.	30°C	7.6	50°C, 20 min	Na^+^, K^+^, Ca^2+^, Mg^2+^	Fe^3+^,Al^3+^,Mn^2+^, Cu^2+^, SDS, EDTA	Zhou et al. (2020)
AlgSH17	*Microbulbifer* sp. SH- 1	30°C	7.0	60°C, 20 min	Mg^2+^, Mn^2+^, Fe^3+^, Co^2+^, Ni^2+^	Ba^2+^, Cu^2+^, Al^3+^	Yang et al. (2021)
Alys1	*Tamlana* sp. s12	35°C	7.0	60°C,20 min	Mg^2+^, glycerol	Ca^2+^, Mn^2+^, Fe^2+^, Fe^3+^, SDS, EDTA	Yin et al. (2021)

**Figure 3:**
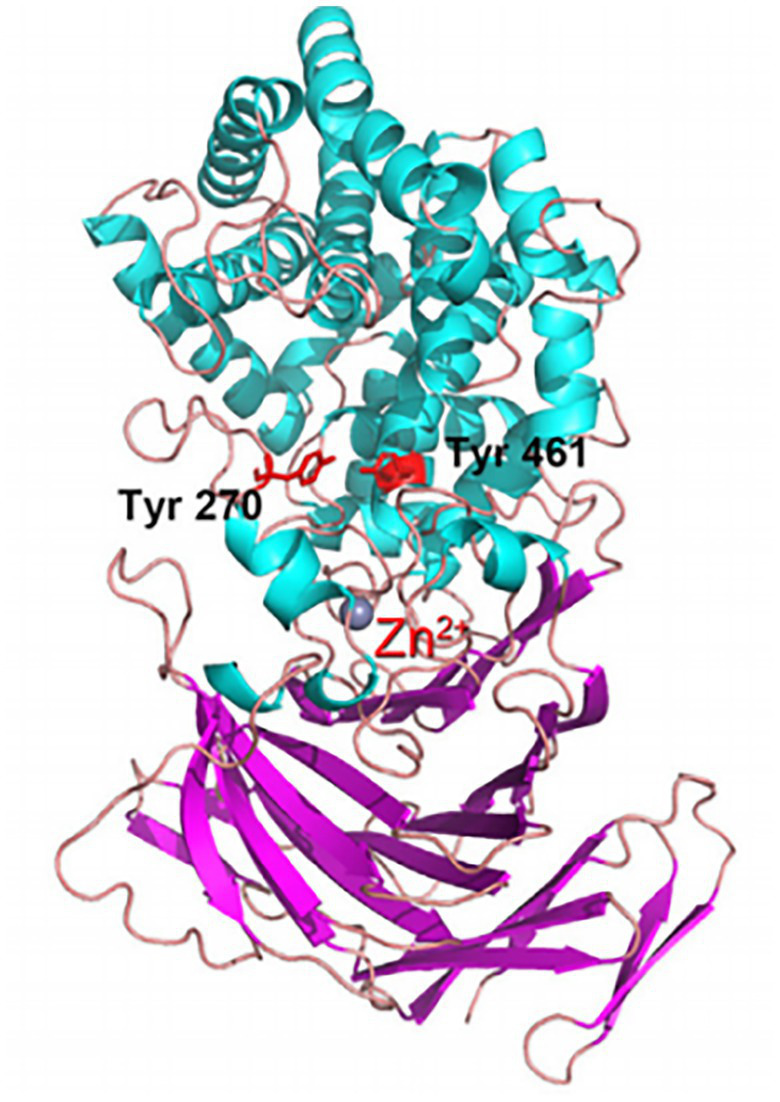
Alginate lyase from yeast *Yarrowia lipolytica* structure (Yang et al., 2021).

Yang et al. (2021) reported the AlgSH17 structure, a cold-adapted alginate lyase with both endolytic and exolytic cleavage activities from *Microbulbifer* sp. SH-1 (Figure 3). The enzyme is composed of two domains. Asparagine, histidine, tyrosine, and arginine jointly constitute the binding pocket, and a Zn^2+^ binding site is near this binding pocket. Consequently, Zn^2+^ competitively binds to the enzyme inhibiting its activity, but Mn^2+^ and Fe^2+^ have activation effects. This research shows that AlgSH17 has good tolerance to some metal ions, suggesting that it has great potential in industrial applications (Yang et al., 2021). Most cold-adapted alginate lyases are neutral enzymes. Enzymatic activity is reduced or even inactivated under hypoacidic or hyperacidic conditions. However, the cold-adapted alginate lyase TAPL7B from *Thalassotalea algicola* has an optimum pH of 11.0, and retains 75% activity at pH 4.0, probably because of the presence of both acidic and basic catalytic domains in its conserved structural fold (Chen et al., 2023).

### 2.4. γ-Glutamylcysteine ligase from cold-adapted synthetase

**Figure 4:**
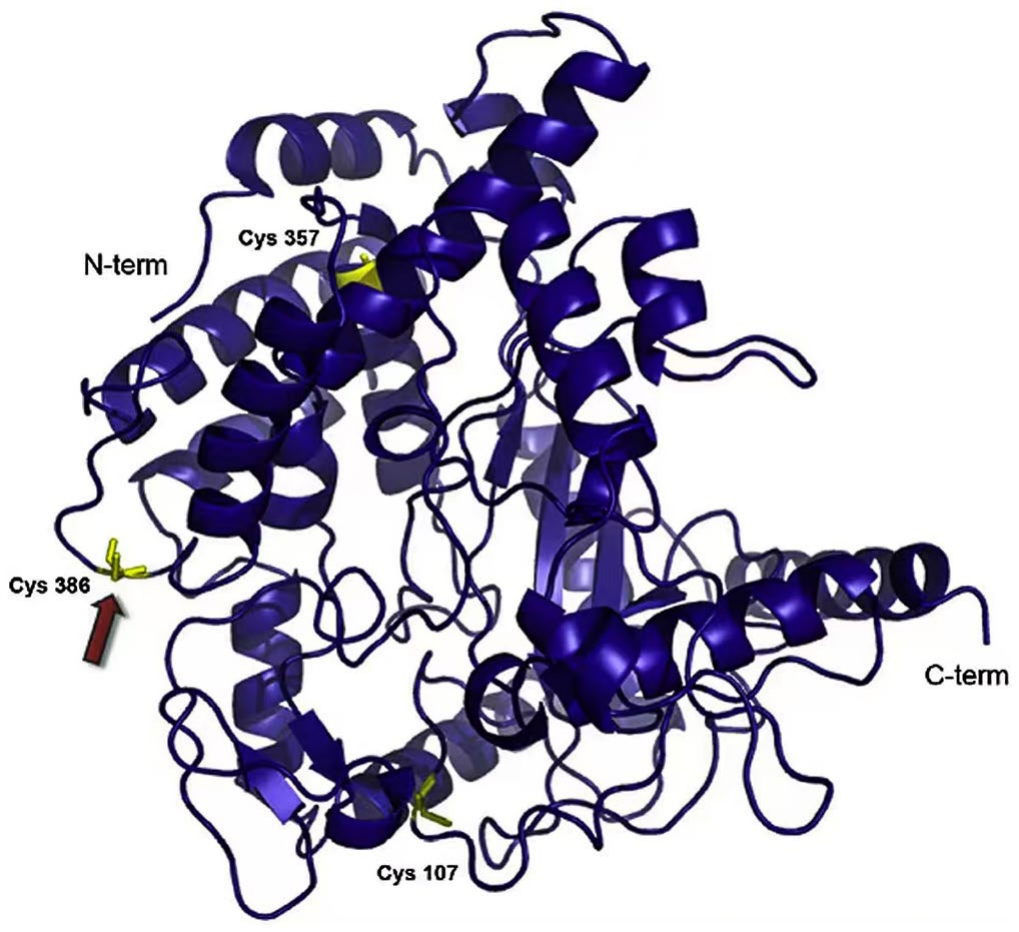
γ-Glutamylcysteine ligase from *Pseudoalteromonas alobanktis* structure (Albino et al., 2014).

DNA ligase, glutathione synthase, and γ-glutamylcysteine ligase are the primary cold-adapted synthetases, with γ-glutamylcysteine ligase being the most often employed (Albino et al., 2012; Munshi et al., 2017). Glutathione synthesized by γ-glutamylcysteine ligase functions in repairing cell damage caused by reactive oxygen species *in vivo*. It is widely used in clinical medicine and has good potential for biopharmaceutical industrial applications (Pallardo et al., 2009; Narayanankutty et al., 2019). Albino et al. (2014) studied the structure and associated phenotype of γ-glutamylcysteine ligase from the psychrophilic bacterium *Pseudoalteromonas haloplanktis* (Figure 4). The enzyme has maximum activity at 15°C and pH 8.0 and is heavily dependent on Mg^2+^. Further analysis of its amino acid sequence and structure found that amino acids without five-membered ring side chain such as cysteine and glutamic acid, and a reduction in the number of disulfide bonds, in its catalytic domain are more conducive to maintaining the enzyme’s structural flexibility (Albino et al., 2014).

### 2.5. Peptidylprolyl isomerase from cold-adapted isomerase

**Figure 5:**
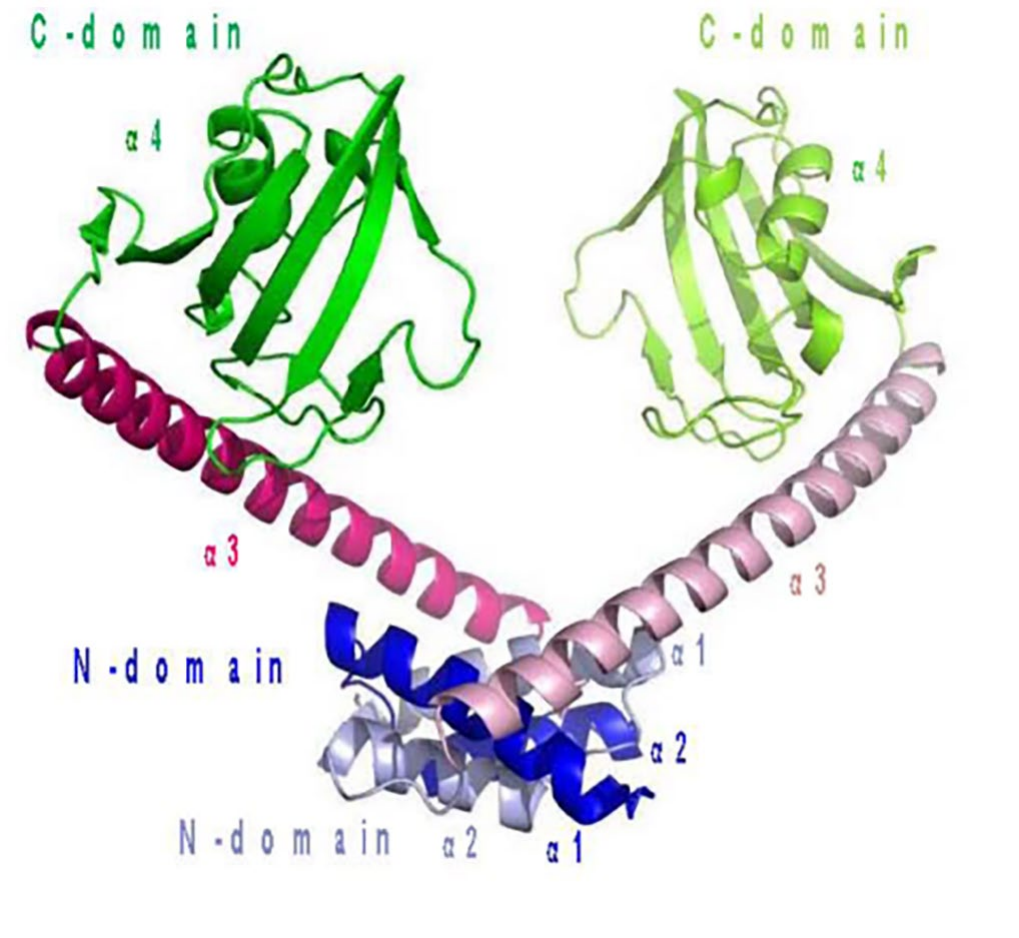
Peptidylprolyl isomerase from *Shewanella* sp. SIB1 structure (Budiman et al., 2011).

Currently, cold-adapted isomerases mainly include cold-adapted phosphotriester isomerase and peptidylprolyl isomerase from *Vibrio* and *Swaniella* sp. SIB1, respectively (Budiman et al., 2011; Aqvist, 2017). *Shewanella* peptidylprolyl isomerase is a folding enzyme that helps intracellular molecular assembly and improves overall low temperature catalytic abilities in this organism. This enzyme, FK506-binding protein 22 (FKBP22) is a typical cold-adapted enzyme, consisting of an N-terminal domain and C-terminal domain, forming a ‘V’ dimer structure, with the N-terminal domain mainly involved in its catalytic reactions (Figure 5). The enzyme’s optimum catalytic temperature is 20°C; therefore, its study helps in the understanding of cold-adapted microorganism catalytic mechanisms overall, and provides a theoretical basis for the improvement of industrial microorganisms microorganism utilization (Budiman et al., 2011).

### 2.6. ATP phosphoribosyl transferase from cold-adapted transferase

**Figure 6:**
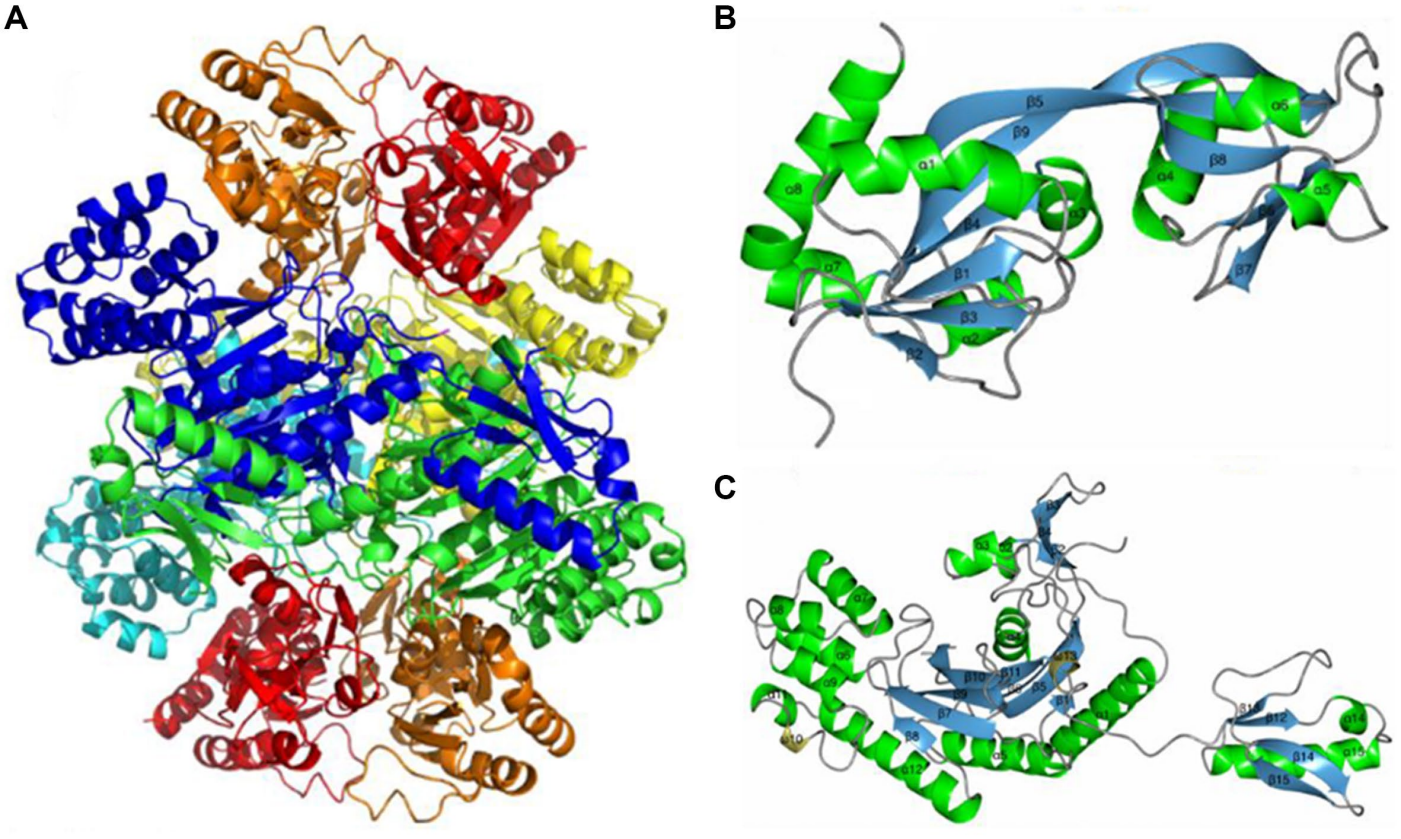
ATP phosphoribosyl transferase from Psychrobacter arcticus structure. **(A)** HisGS and HisZ heterooctamer structure; **(B)** HisGS monomer structure; **(C)** HisZ monomer structure (Stroek et al., 2017).

ATP phosphoribosyl transferase is representative and the most commonly reported cold-adapted transferase. Two ATP phosphoribosyl transferase subfamilies from *Thermotoga maritima* and *Lactococcus lactis* are descriptive. One is HisG_L_ which contains a catalytic domain, and the other is HisG_S_, which does not and needs to bind HisZ to function (Champagne et al., 2005; Vega et al., 2005). Subsequently, Stroek et al. (2017) analyzed the crystal structure of an HisG_S_ and HisZ complex from the psychrophilic polar bacterium *Psychrobacter arcticus* (Figure 6), and found that the existence of HisZ made HisG_S_ more sensitive to temperature rise, and that the flexibility of its structure increased after the combination of HisZ and HisG_S_. Unlike the cold-adapted enzymes mentioned above, this enzyme may increase the contact surface of the enzyme with the solvent *via* HisZ and HisG_S_ binding, thereby increasing enzyme flexibility, which in turn increases substrate affinity and enzymatic reaction rates of at low temperatures (Isaksen et al., 2016).

## 3. Microorganism cold-adapted enzyme applications

Cold-adapted enzymes have numerous industrial benefits. These include easily managed reaction conditions, easy monitoring and control of the production processes, and a wide variety of commercial applications. Therefore, these enzymes are widely studied and have been applied in various areas, including food processing, detergent production, bioremediation, environmental protection, straw resourcing, and in basic molecular biology research (Cavicchioli et al., 2011; Yadav et al., 2022).

### 3.1. Applications in food processing

Cold-adapted enzymes are often used in the food processing industry to help maintain the nutritional value and flavor of particular ingredients during processing and treatment, and to replace mesophilic or thermophilic enzymes in the processing and treatment of food materials. Representative examples include cold-adapted β-galactosidases, esterases, xylanases, and proteases (Kumar et al., 2021).

β-galactosidase can hydrolyze the lactose in dairy products, which effectively resolves the issue of human lactose sensitivity in dairy products (Ugidos-Rodriguez et al., 2018). However, dairy product treatment with β-galactosidase combined with pasteurization or ultra-high temperature sterilization is not conducive to preserving the taste and nutrition of dairy products (Krishna et al., 2021). In contrast, the cold-adapted β-galactosidase BsGal1332, can hydrolyze lactose at 0°C and loses its activity at 50°C (Liu et al., 2021). These special properties may effectively solve the nutrient loss problem caused by conventional lactose treatment in dairy products.

Esterase can maintain the characteristic flavor of cheese during cheese ripening, but mesophilic and higher temperature-adapted esterases can decompose certain amino acids causing a loss of flavor during the usual higher temperatures required for dry cheese fermentation. Whereas a cold-adapted esterase from *Lactobacillus plantarum* can catalyze at 5°C is inactivate after 30 min at 55°C, and has some acid and salt resistance, and therefore, can be used as an exogenous esterase to maintain cheese flavor in fully ripened cheeses (Esteban-Torres et al., 2014).

The addition of xylanase can improve the water holding capacity and improve the quality of wheat bread, but thermophilic xylanase can cause the excessive release of water and gelatinization during baking (Jiang et al., 2005). Li et al. (2022) found that a recombinant cold-adapted xylanase originally from *Sorangium cellulosum* (XYNSC8) at a low dose (0.05–0.2 mg/kg flour) can effectively improve the quality of bread, and its effect was obviously better than that of mesophilic xylanases available on the market.

Proteases can improve the tenderness of meat while processing meat products (Mangiagalli et al., 2020). Currently, commercial meat tenderizer proteases such as papain are mesophilic or thermophilic. Zhao et al. (2012) studied a cold-adapted collagenolytic enzyme from *Pseudoalteromonas* (MCP-01) and found that it can selectively degrade muscle fiber protein at 4°C, improving beef tenderness, while keeping meat quality and color.

### 3.2. Applications in the detergent industry

In traditional laundry applications, chemical detergents are often stirred at high temperatures with fabrics to remove stains. But long-term stirring at high temperatures reduces the lifespan of clothing, and also increases energy consumption. However, adding an enzyme preparation to the detergent can produce ideal washing effects in more gentle conditions (Kuddus and Ahmad, 2012; Sarmiento et al., 2015). Proteases can decompose adsorbed protein residues into small molecular peptides, consequently dissolving the residue into detergent laden water, thus removing protein stains. Unfortunately, mesophilic and thermophilic proteases have low activity at low temperatures, so it is difficult to obtain the ideal benefits of cold water washing (Kuddus and Ramteke, 2009).

The cold-adapted alkaline protease of *Stenotrophomonas maltophilia* has good activity at 20°C, high stability, good compatibility with commercial detergents, and good stain removal at low temperatures. Therefore, it can be added to commercial detergents to remove protein-containing stains such as milk and blood (Mohammed and Pramod, 2011).

Adding lipases to detergents can effectively remove grease, but the maximal activity temperature of commonly used lipases is generally between 30°C and 50°C. Adding mesophilic or thermophilic lipases to detergents not only increases energy consumption, but also increases garment damage due to high temperature washing. The cold-adapted lipase “Lipoclean” produced by Novozymes can remove lipid substances at low temperatures. When mixed with detergent this enzyme can even achieve good cleaning effects on porous building materials (Mhetras et al., 2021).

Cold-adapted amylases can effectively remove starch-containing stains such as chocolate and tomato sauce. Adding *Pseudoalteromonas* Amy175 can greatly improve the ability of commercial detergents to remove stains (Wang Y. et al., 2018; Wang X. et al., 2018). The DuPont Bioscience Company developed “PREFERENZ” cold-adapted amylase to add todetergent. It has a high activity at 16°C and effectively removes starch stains (Edser, 2004).

### 3.3. Applications in bioremediation and environmental protection

Soil and water pollution are currently major problems worldwide. Antibiotic and dye pollution in soils and water resources seriously affects ecology and accumulates in heterotrophic bodies through the food chain, including hemans, resulting in irreversible harm. Cold-adapted laccase can degrade aromatic compounds, including most antibiotics under mild conditions. Unlike mesophilic and thermophilic laccase, cold-adapted laccase can be inactivated immediately at a lower temperature (40°C) after a reaction, without affecting subsequent reactions. The degradation rate of crystal violet using a coupling system of cold-adapted laccase from *Kabatiella bupleuri* G3 IBMiP and 2,2′-azino-bis (3-ethylbenzothiazoline-6-sulfonic acid) diammonium salt (ABTS) can reach 48% (Wiśniewska et al., 2021). Tetracycline and oxytetracycline can be degraded effectively at 0°C with a cold-adapted laccase-ABTS coupling system from the white-rot fungus *Pycnoporus* sp. SYBC-L10 (Tian et al., 2020).

As demand for fossil fuels grows, oil spills are an increasingly greater threat to the environment. The cold-adapted lipase produced by *Bacillus cereus* HSS can effectively remove oil from wastewater; using sponge immobilized cells, it can achieve an oil removal elimination efficiency of 94.7% (Hassan et al., 2018). Another good choice for the treatment of waste oil is to produce biodiesel. Novozymes produces a cold-adapted lipase named “Novozyme 435” that can be used convert waste oil into biodiesel (Mhetras et al., 2021). Unfortunately, “Novozyme 435” is not stable in high methanol concentration environments. However, the cold-adapted lipase M37 from *Photobacterium lipolyticum* retains high activity in a methanol concentration of 10% and can be used to produce biodiesel (Yang et al., 2009). M37 showed significantly higher stability and conversion rates than “Novozyme 435” in both one and two methanol feed reactions demonstrating good application prospects in waste oil biodiesel production.

### 3.4. Applications in molecular biology

Gentle thermal treatment of cold-adapted enzymes causes irreversible enzymatic inactivation without interfering with subsequent reactions during *in vitro* molecular biology. Moran et al. (2001) isolated a strain of psychrophilic bacteria from seawater that secreted a cold-adapted protease (A9) that could digest restriction endonuclease PvuII to prevent it from cleaving DNA without interfering with DNA quality. However, the enzyme can also interfere with Taq polymerase activity, so A9 protease can be added to a reaction, then the enzyme can be inactivated to terminate its reaction under mild thermal conditions and ensure the subsequent reactions are not interfered with (Moran et al., 2001).

Alkaline phosphatase dephosphorylates DNA and is often used to clean up PCR products and prevent multiple cycles before cloning, but persistence of alkaline phosphatase activity can interfere with subsequent reactions. Most commercially available alkaline phosphatases are mesophilic enzymes, which generally require medium-high inactivation temperatures. This can lead to DNA degradation and affect nucleic acid quality. However, alkaline phosphatase from the Antarctic psychrophilic bacterium TAB5 can be inactivated after incubation at 50°C for 20 min. Inactivating the phosphatase at this temperature does not affect the quality of the sample (Rina et al., 2000).

DNA ligase catalyzes the ligature of DNA fragments by phosphodiester bonds. When used *in vitro* in molecular biology it is usually derived from a bacteriophage and has optimal activity at a fairly low temperature. The cold-adapted T4 DNA Ligase of Takara Bioscience Company is widely used in research (Kumar et al., 2021). Berg et al. (2019) isolated three cold-adapted ATP-dependent DNA ligases from psychrophilic bacteria. Among them, Vib-Lig from *Aliivibrio salmonicida* retained 60% of its activity at 20°C and 80% of its substrate bonding efficiency at 10°C, demonstrating good application prospects for basic research.

### 3.5. Applications for straw resourcing

Making full use of biomass such as agricultural waste to produce ethanol helps solve environmental pollution problems caused by agricultural waste and help ease energy shortage problems. A primary agriculture waste is straw. However, the conversion efficiency of lignocellulose to fermentable sugars is low and the cost is high because of straw’s structure, which limits the large-scale industrialization of cellulosic ethanol. Currently, optimal activity temperature of the enzymes, xylanase, and cellulase, associated with straw degradation is about 55°C. Normal environmental temperatures do not meet the requirements of straw degradation enzymes (Wang et al., 2021). Thus, the development of cold-adapted enzymes to degrade straw is central to the overall utilization of straw resources. Sun et al. (2018) expressed and purified a β-glucosidase from a marine bacterium with maximal activity at 25°C and pH 6–8 that is rapidly inactivated after incubation at 40°C. The enzyme is not affected by NaCl nor organic solvents, and the activity of the enzyme can be increased in the presence of reducing agents. These cold-adapted characteristics provide great potential for the large-scale production of cellulosic ethanol at room temperature. Cold-adapted laccases, xylanases, and β-xylosidases also have great potential in the industrial application of straw degradation to produce cellulose ethanol (Kim et al., 2021). Owing to the particular composition of straw, the effect of a single enzyme is not evident in its breakdown. The synergistic synergistic effect of the *Cladosporium cladosporioides* cold-adapted catalytic enzyme system for lignocellulose degradation with commercial xylanase is impressive. Having maximal activity at 28°C, and a sugar yield of 10.1 mg/ml, this method can greatly reduce the cost of straw conversion to ethanol (Ji et al., 2014).

With the development of modern biotechnology, cold-adapted enzymes are playing a major role in an increasing number of fields. Representative cold-adapted enzymes of all types different microorganisms with associated application potentials are summarized in Table 4.

**Table 4:** Cold-adapted enzymes from various microorganisms and associated applications.

Enzyme	Microorganism	Source	Field of application	References
DNA Ligase	*Pseudoalteromonas haloplanktis*	Deep sea	basic Biology Research	Georlette et al. (2000)
Glucose Oxidase	*Cladosporium neopsychrotolerans* SL16	Alpine soil	feed production, pharmaceutical technology, food industry	Ge et al. (2020)
Protease	*Janthinobacterium licidum*	Polar soil	detergent production, food industry, pharmaceutical process, environmental bioremediation	Kim et al. (2018)
Alginate Lyase	*Vibrio* sp. W2	Deep sea	food industry, clinical medicine, basic biological research	Wang Z. P. et al. (2020); Wang Q. et al. (2020)
Chitosanase CsnS	*Serratia* sp. QD07	Deep sea	clinical medicine, industrial production	Zheng et al. (2021)
β-1,4-glucanase	*citrisub* sp.	Mountain plant pathogens	food industry	de Melo et al. (2021)
Glucoamylase	*Tetracladium* sp.	Polar soil	Biofuels, food industry	Carrasco et al. (2017)
Pullulanase	*Bacillus methanolicus* PB1	Polar soil	food industry	Zhang et al. (2020)
α-Amylase	*Arthrobacter agilis*	Polar soil	Food industry, basic biology research	Kim et al. (2017)
Dextranase	*Catenovulum* sp. DP03	Deep sea	food industry	Deng et al. (2020)
Lipase	*Pseudomonas* sp. KE38	Deep sea	Food Industry, detergent production, environmental bioremediation	Salwoom et al. (2019)
Superoxide Dismutase	*Paelopatides* sp.	Deep sea	Cosmetic production, clinical medicine research	Li et al. (2019)
FK506-Binding Protein	*Shewanella* sp.	Deep sea	Basic biological research	Budiman et al. (2011)

## 4. Mechanisms for cold-adapted catalysis in cold-adapted enzymes

Cold-adapted enzymes usually enhance the affinity of the enzyme substrate by reducing the amount of activation energy needed for the enzyme-substrate complex. This is accomplished by increasing the flexibility of all or part of the enzyme’s structure, which increases the rate of the enzymatic reaction at low temperatures, consequently reducing energy consumption. Presently, the catalytic mechanism of cold-adapted enzymes is generally explained in two respects: (1) The structure is different from that of a mesophilic or thermophilic enzyme possessing greater structural flexibility. (2) The enzyme has a lower activation energy level, such that enzyme molecule energy consumption is low in low temperature conditions (Cavicchioli et al., 2011; Siddiqui, 2015).

### 4.1. Cold-adapted enzymes structural flexibility

Structurally, compared with the mesophilic and thermophilic enzymes, cold-adapted enzymes have obvious differences in the number of glycine, arginine, and proline residues, as well as in the number of salt bridges, hydrogen bonds, disulfide bonds, and overall hydrophobicity and hydrophobic surfaces. The number of proline residues, salt bridges, hydrogen bonds, and disulfide bonds are decreased, and an increase in the number of glycine residues and in overall hydrophobicity and hydrophobic surfaces are common features of most cold-adapted enzymes (Siddiqui and Cavicchioli, 2006).

#### 4.1.1. Primary structure

**Figure 7:**
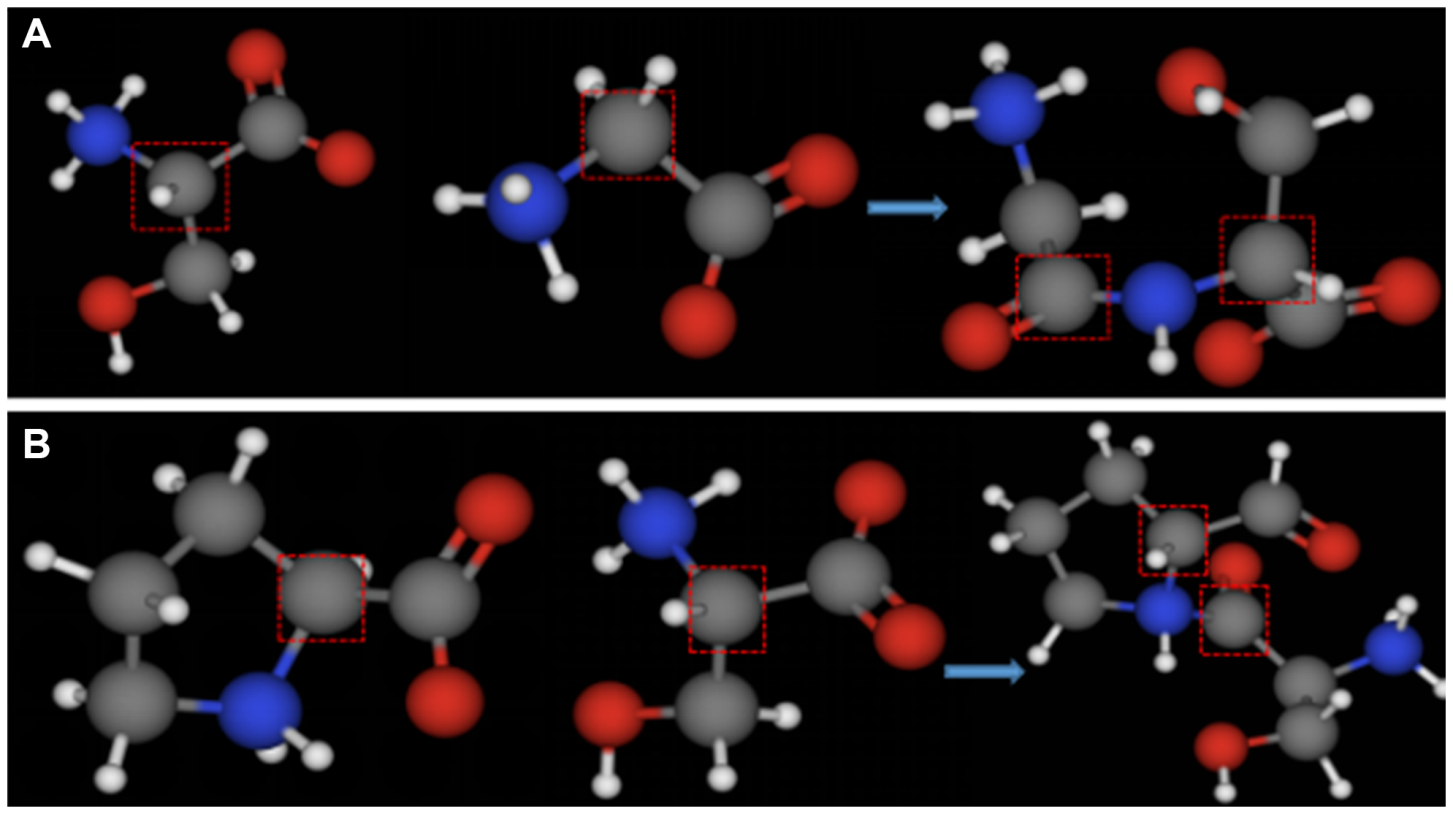
Ball-and-stick models of the dipeptide formed by the condensation of different amino acids. **(A)** Ball-and-stick model of serine and glycine dipeptide; **(B)** ball-and-stick model of proline and serine dipeptide (the gray part in the red box represents the main chain carbon atom of each amino acid).

Cold-adapted enzyme amino acid sequence analysis shows that the number of proline residues in cold-adapted enzymes is much lower than that in mesophilic and thermophilic enzymes (Wang Y. et al., 2018; Wang X. et al., 2018; Hou et al., 2019). Proline’s α-carbon atom is connected to its five-membered ring structure side chain group, which makes it difficult for the main chain carbon atom to rotate This limits the rotation of the carbon main chain during the formation of a peptide chain (Figure 7B), increases the rigidity of the peptide chain, and reduces local flexibility of the protein, but to a certain extent, it improves the stability of a protein, namely the ‘proline law’ (Watanabe et al., 1996). Studies on the homologs of mesophilic and thermophilic oligosaccharidases show that proline content of high temperature oligosaccharidases is significantly higher than that of mesophilic homologs (Land et al., 2019). The absence of proline increases the flexibility of a cold enzyme structure, thus facilitating attachment to its substrate. Additionally, the presence of cysteine can increase the stability of proteins by generating disulfide bonds during the formation of higher structure (Aghajari et al., 1998). The effect of disulfide bonds on protein stability will be discussed further below.

Glycine, serine, and histidine are often found in the central domain of cold-adapted enzymes (Mahdavi et al., 2011; Raymond-Bouchard et al., 2018). This may be because no amino acid has a five-membered ring side chain structure other than proline. Therefore, substitution by these amino acids reduces restriction on the rotation of the main chain (Figure 7A), thus increasing molecular conformation flexibility (Raymond-Bouchard et al., 2018). The proportion of amino acids with simple side chains in the primary structure of cold-adapted enzymes determines the flexibility of the structure. However, increased structural flexibility inevitably leads to reduced thermal stability. Therefore, in the modification of cold-adapted enzymes, proline is often used as an alternative to other amino acids to improve the stability of cold-adapted biomodified enzymes (Xu et al., 2020).

Arginine’s structure allows it to form five hydrogen bonds and two salt bridges in tertiary structure with other amino acid residues. The existence of hydrogen bonds and salt bridges rendera protein structure more stable. Nonetheless, in most cold-adapted enzymes, the level of arginine residues on the surface of the protein is often higher than that of mesophilic or thermophilic homologs (Zheng et al., 2016). The presence of arginine on the structural surface of cold-adapted enzymes may facilitate interaction with solvents instead of forming hydrogen bonds and salt bridges with other residues on that protein surface, actually contributing more flexibility to the molecular structure of cold-adapted enzymes (Siddiqui and Cavicchioli, 2006).

#### 4.1.2. Advanced structure and surface properties

Hydrogen bonding plays an important role in protein folding, and a main driving force of protein folding is the formation of hydrophobic cores (Lee et al., 2017). A reduction in the number of hydrogen bonds leads to a sharp decrease in the hydrophobicity of an enzyme, but at the same time, increases its flexibility parameters to some extent (Bentahir et al., 2000). Furthermore, the existence of amino acid residues such as arginine and glutamic acid in enzyme structure may lead to the formation of salt bridges when an enzyme molecule interacts with substrate molecules. Although these salt bridges are non-covalent interactions with weak binding force, the existence of a large number of these amino acids will produce more salt bridges, which will increase the stability yet reduce the flexibility of an enzyme molecule. This is why the salt bridge content in the structure of cold-adapted enzymes is lower than that of homologous mesophilic enzymes (Wang et al., 2016).

The disulfide bond is a stable covalent bond that can make protein structures more complex and enhance protein stability under certain conditions (Siddiqui et al., 2005). The Cys70-Cys115 disulfide bond is unique to mammalian α-amylase. The number of disulfide bonds decreases gradually in thermophilic, mesophilic, and psychrophilic α-amylase, and the Cys70-Cys115 disulfide bond is always absent in the cold-adapted α-amylase produced by low temperature microorganisms (Janecek, 1997; Aghajari et al., 1998). Disulfide bonds often form between peptide chains over much of the molecule, so the absence of disulfide bonds leads to a decrease in the thermal stability of an enzyme while increasing the flexibility of the molecule (Siddiqui et al., 2005). Protein folding also affects the cold catalytic activity of cold-adapted enzymes. Some enzymes have similar amino acid sequences yet possess different thermodynamic effects. The weakening of interactions between N-terminal, C-terminal, and various internal domains all affects the flexibility and stability of an enzyme (Feller et al., 1999).

Hydrophobicity and hydrophobic surface area also receive particular attention in the study of cold-adapted enzyme structure and mechanism. Hydrophobic pockets in an enzyme molecule can reduce the interference of water in an enzymatic reaction, facilitate enzymatic reaction transition states, and reduce the activation energy of an enzymatic reaction, thereby reducing the energy consumption of an enzymatic reaction at low temperature. An analysis of many cold-adapted enzyme structures found that the overall hydrophobicity and number of hydrophobic surfaces of cold-adapted enzymes is significantly higher than that of homologous mesophilic or thermophilic enzymes (Zheng et al., 2016; Rutkiewicz et al., 2019). This confirms that the existence of hydrophobic pockets enables cold-adapted enzymes to catalyze reactions at lower temperature more easily.

### 4.2. Activation energy and cold catalysis of cold-adapted enzymes

In severely low temperature conditions, the main problem faced by cold-adapted enzymes is maintaining normal catalytic function. The catalytic efficiency of a cold-adapted enzyme is higher and the activation energy lower than that of mesophilic and thermophilic enzymes.

According to the Arrhenius equation:

(1) $$k_{\mathrm{cat}}=Ake^{-E_{a}/RT}$$

where “*A*” represents an exponential factor; “*k*” is the dynamic transfer coefficient (generally 1); “*E*_a_” is the activation energy; “*R*” is the gas constant; and “*T*” is the absolute temperature. Reaction rates decrease sharply as temperature decreases. Furthermore, the viscosity of culture medium increases and the molecular diffusion rate decreases at low temperature, which also leads to a decrease in the reaction rate, that is, low temperatures influence the smooth progress of an enzymatic reaction (Siddiqui and Cavicchioli, 2006). In this case, the value of dynamic transfer coefficient “*k*” is not set to 1. Based on mean viscosity and transition state theory, the following results are obtained:

(2) $$k_{\mathrm{cat}}=\sqrt{1+\left(\eta^{\prime }/\eta_{1}\right)}-\left(\eta^{\prime }/\eta_{1}\right)$$

wherein, “*η*” is relative viscosity, and “*η*_1_” is an unknown parameter that is determined according to experimental fitting. When “*η*_1_” is greater than or equal to 10, the value “*k*” is close to 1 and can be obtained from Equation 1 and transitional state theory:

(3) $$k_{\mathrm{cat}}=\left(K_{B}T/h\right)e^{-\Delta G/RT}$$

Here “*k*_cat_” is the enzymatic reaction rate; “*K*_B_” is the Boltzmann constant (~1.38 × 10^−23^ J·K^−1^); “*h*” is Planck constant (~6.63 × 10^−34^ J·Hz^−1^), and “Δ*G*” is the activation energy.

The rate of an enzymatic reaction can be increased by lowering Δ*G*. This can be achieved by stabilizing an activated substrate in a transitional state or by destabilizing an enzyme–substrate complex (increasing the Michaelis constant *k*_m_). Cold-adapted enzymes reduce activation energy at low temperatures by increasing the turnover number (*k*_cat_) or by increasing enzyme–substrate complex affinity. This maintains reaction equilibrium and overcomes chemical reaction rate decreases caused by low temperatures (Zanphorlin et al., 2016).

The *k*_cat_/*k*_m_ value can be used to measure the catalytic efficiency of an enzyme and the affinity of an enzyme to its substrate. Sometimes an increase in catalytic rate indicates that the activation energy of an enzyme is lower than that of other enzymes. Iyo and Forsberg (1999) compared the *k*_cat_/*k*_m_ of two endoglucanases from *Fibrobacter succinogenes* S85 at different temperatures CelG was designated the cold-adapted version. The *k*_cat_/*k*_m_ of CelG was 75 times and 10 times higher than that of the other endoglucanase at 4°C and 25°C, respectively, and the catalytic efficiency of CelG was also higher at these lower temperatures (Iyo and Forsberg, 1999; Table 5). Under similar low temperature conditions, the higher the *k*_cat_/*k*_m_ value, the lower the activation energy. The activation energy of cold-adapted enzymes is lower than that of mesophilic or thermophilic enzymes, and this lower activation energy reduces the energy consumption of cold-adapted enzyme catalytic reactions.

**Table 5:** Kinetic parameters of Fibrobacter succinogenes S85 CelG and EGD (Iyo and Forsberg, 1999).

Enzyme	Temperature	Kinetic parameter		
		*K*_cat_ (s^−1^)	*K*_m_ (mg/mL)	*K*_cat_/*K*_m_
CelG	4	20.1	6.8	3.0
	25	93	55	1.7
EGD	4	0.6	16.1	0.04
	25	4.0	23.8	0.17

Enzyme molecules can assume a variety of different conformations, in which natural conformations dominate while alternate conformations can be transformed from one to another. An increase in the flexibility of an enzyme molecule can increase its number of conformational isomers, which relates to the catalytic function of the enzyme. This is because increased flexibility reduces energy consumption required for the induction of the enzyme to bind to the substrate and makes control of the isomer easier to achieve (Zecchinon et al., 2001).

Zavodsky et al. used infrared spectroscopy to detect the exchange of hydrogen and heavy hydrogen in two 3-isopropyl malate dehydrogenases, one mesophilic, one thermophilic. By comparing the relative flexibility of the two 3-isopropyl malate dehydrogenases, they found that under the most appropriate catalytic conditions, the two 3-isopropyl malate dehydrogenase structures have similar flexibility. However at room temperature, the mesophilic enzymes has a looser molecular structure compared with the thermophilic counterpart (Zavodszky et al., 1998). That is, thermophilic enzymes are less efficient at room temperature, perhaps because a more closed conformation at room temperature hinders the interactions in between enzyme-substrate complexes. In contrast, mesophilic or cold-adapted enzymes have higher conformational flexibility at room or low temperatures. These enzymes bind to substrates easier consuming less energy in the process which subsequently reduces the activation energy required for catalytic reaction and improves over efficiency.

To investigate relationships between cold-adapted enzyme structure and enzymatic reaction at cryogenic temperatures, several researchers have compared the crystal structure of a variety of cold-adapted enzymes with that of homologous mesophilic or thermophilic enzymes. Trypsin, citric acid synthase, α-amylase, and malate dehydrogenase are representative of those studied. The active sites of the cold-adapted enzymes all possess a larger cavity than homologous mesophilic and thermophilic counterparts. Therefore substrates are more easily brought closer together, thus reducing the activation energy required for enzyme-substrate complex formation. However, large active cavities may cause a reduction in enzyme-substrate affinity. This means that cold-adapted enzymes can increase the probability of complex enzyme-substrate formation by possessing a larger active site cavity, thereby increasing the rate of enzymatic reaction (Smalas et al., 1994; Russell et al., 1998; Kim et al., 1999; Aghajari et al., 2002; Voigt et al., 2002).

**Figure 8:**
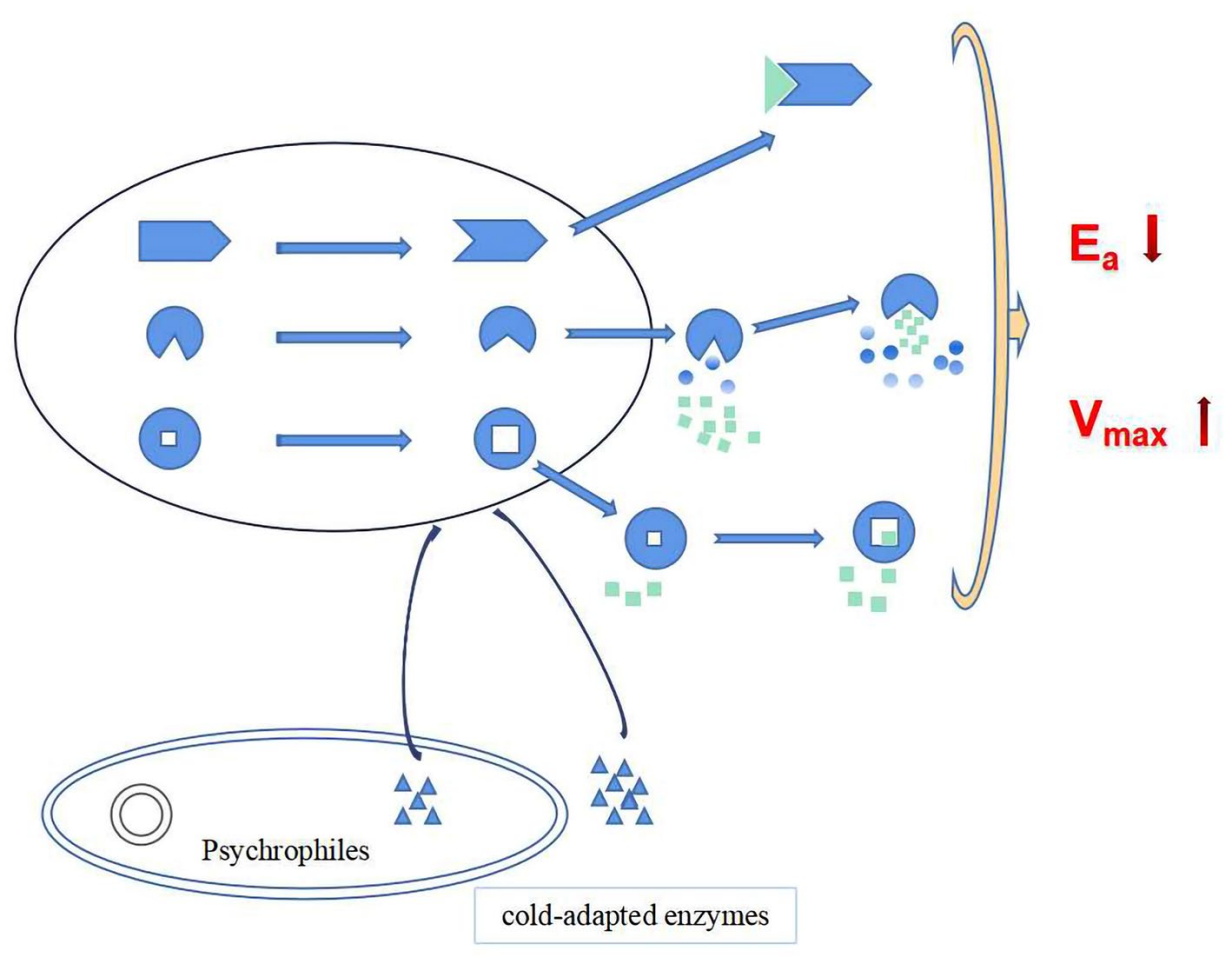
Cold catalytic mechanism of cold-adapted enzymes (green square and triangle as substrate molecules, gradient color round as water molecules).

In summary, at low temperatures, cold-adapted enzymes ensure the smooth progress of catalytic reactions through the following three strategies: (1) Flexibility of the structural domain in the molecule is increased which increases the affinity of the enzyme-substrate complex, thereby reducing the activation energy required by the chemical reaction and ensuring the smooth progress of the catalytic reaction at low temperature. (2) Hydrophobic surface area within the hydrophobic pocket is increased, which reduces the interference of water on the enzymatic reaction thus the enzymatic reaction more easily to forms a transition state, t the activation energy required by the enzymatic reaction is reduced, and energy consumption of the enzymatic reaction at low temperature is reduced. (3) Formation of a larger cavity in the active site increases the probability for the formation of an enzyme-substrate complex, thereby increasing the enzymatic reaction rate (Figure 8; Mangiagalli and Lotti, 2021).

## 5. Cold-adapted enzyme molecular modification

Cold-adapted enzymes can catalyze biological reactions at low temperatures. These enzymes have the advantage of requiring low temperatures to achieve activation, and so are widely used in industry. However, owing to inherent structural characteristics, naturally low-temperature enzymes have greater sensitivity to pH, metal ions, and organic solvents, which confer the characteristics of low thermodynamic stability, and easy inactivation. Furthermore the reaction condition requirements of cold-adapted enzymes are often difficult to meet in industrial production processes resulting in enzymatic inactivation. Therefore, the molecular modification of cold-adapted enzymes has become a hot topic (Shemsi et al., 2019). Molecular modification of naturally cold-adapted enzymes can be achieved using rational or irrational design.

### 5.1. Irrational design

**Figure 9:**
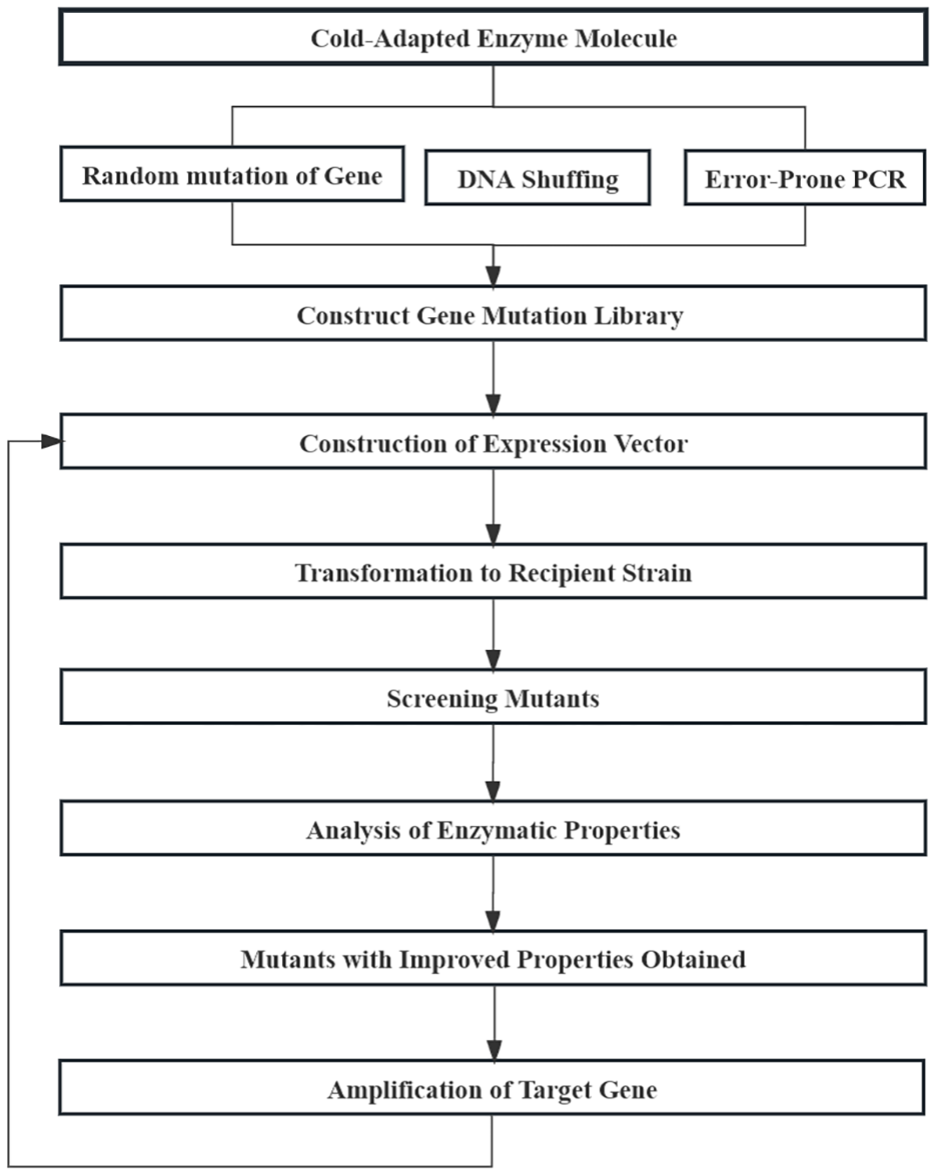
Basic process of enzyme molecules irrational design.

Irrational design of enzyme molecules is also known as directed evolution. Using known structural information and the catalytic mechanism of enzyme molecules, by simulating random mutation, and by using recombination and other natural evolutionary mechanisms, directional pressure can be exerted in later screening process to obtain desired mutants with excellent performance (Jackel et al., 2008). Irrational design needs to construct an efficient mutant library and screening system using error-prone PCR, DNA shuffling, or other technologies to obtain target enzyme molecules (Figure 9; Yang et al., 2014). LipC, a cold-catalyzed lipase derived from a psychrophilic strain of *Pseudomonas aeruginosa*, is a cold-acting lipase that tolerates elevated concentrations of ions and heavy metals. The enzyme has strong substrate specificity at elevated temperatures but loses stability at those temperatures. Cesarini et al. (2012) constructed eight LipC mutant libraries with two point mutations using error-prone PCR technology and then screened for a mutant with both cold catalytic properties and thermal stability from more than 3,000 mutant clones.

DNA shuffling technology can obtain a large number of mutations through *in vitro* recombination. Wintrode et al. (2001) performed eight cycles of mutation recombination on subtilisin S41 using DNA shuffling technology, and inactivation temperatures of an obtained mutant increased by 25°C. Furthermore, constructing mutant libraries using multi-technology is common in the irrational modification of cold-adapted enzymes. Arrizubieta and Polaina (2000) combined error-prone PCR and DNA shuffling technology to increase the half-life of *Paenibacillus polymyxa* β-galactosidase by 20 times at 55°C. The irrational design of enzymatic molecules does not target a specific targeted modification, rather it is random. Therefore, owing to its large workload and low probability of obtaining positive mutants, it is seldom applied to the modification of cold-adapted enzymes.

### 5.2. Rational design

**Figure 10:**
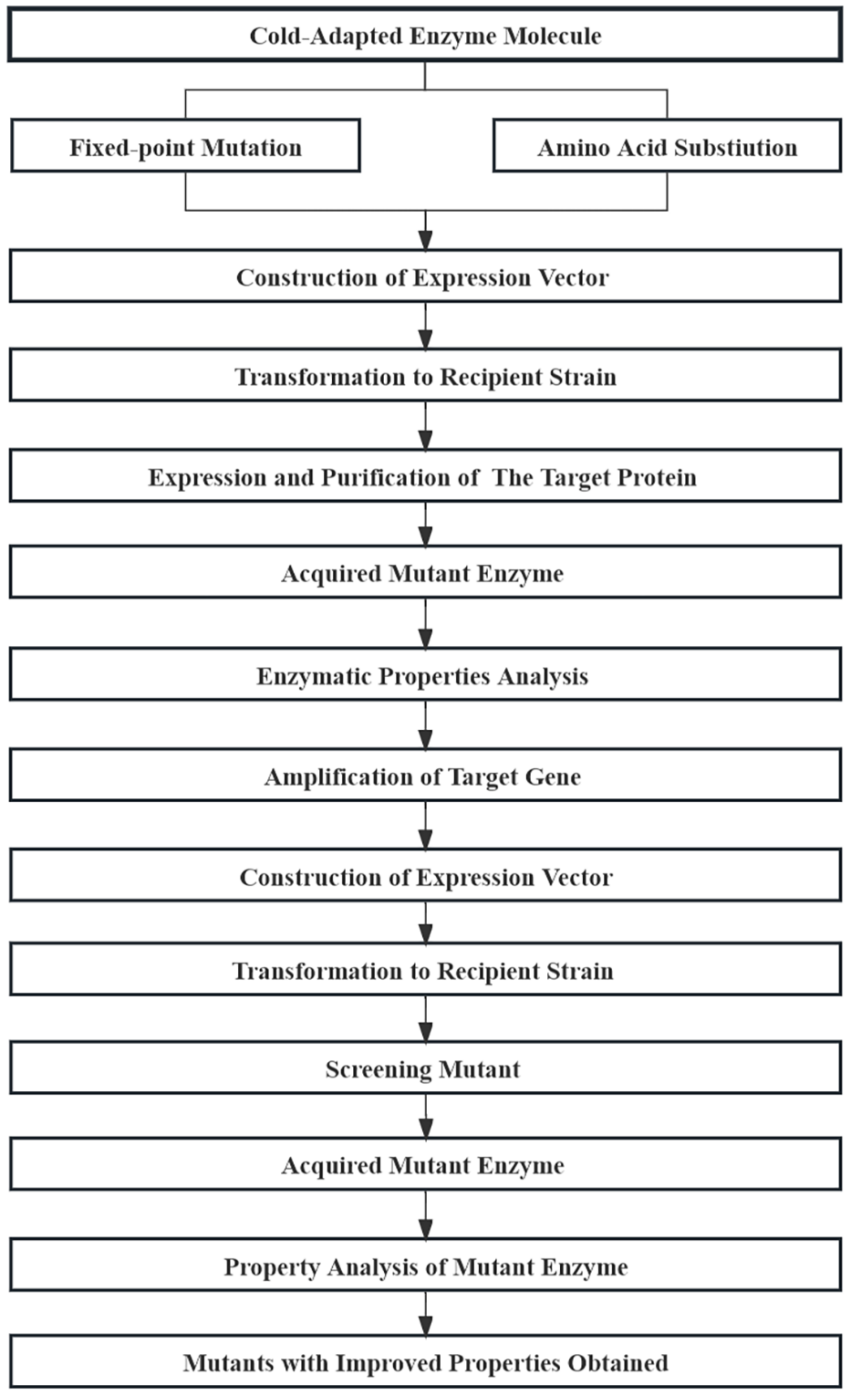
Basic process of enzyme molecule rational design.

Rational design attempts to derive and design a specific amino acid sequence that corresponds to a specific function based on the secondary and tertiary structure of representative enzymatic molecules. Site-directed mutagenesis is used to achieve the amino acid substitutions necessary for the desired structure (Figure 10; Oskarsson and Kristjansson, 2019; Oskarsson et al., 2020). Several strategies, including homologous sequence alignment, disulfide bond-based design, and SCHEMA simulation, have been used to improve the thermostability of cold-adapted enzymes (Voigt et al., 2002; Veno et al., 2019).

Chang et al. (2016) analyzed the pullulanase sequence of *Bacillus naganoensis* using homologous sequence alignment. Six site-directed mutants were obtained and three screened positive for higher thermostability after mutagenesis. Thermal stability is an ideal characteristic for the preservation and application of various cold-adapted lipases. The thermal stability of a cold-adapted lipase can be improved by introducing a cysteine residue into the “cap region” of a cold-adapted lipase (Veno et al., 2019). According to the proline law, Yang et al. (2017) introduced proline residues into the A and B domains of the surface loop of α-amylase from the Antarctic ciliated protozoon *Euplotes focardii* (EfAmy), which greatly improved the thermostability and catalytic efficiency of the enzyme. Presently, most enzyme molecule modifications rely on the crystal structure of that enzyme or homologous molecules and ignore the influence of enzymatic molecular dynamics. Li et al. (2020) combined molecular dynamics simulations and energy optimization methods to design unconserved residue mutants of cold-adapted α-amylase (PHA) from *Pseudoalteromonas haloplanktis* and was able to obtain double mutants with higher thermal stability than the native enzyme.

## 6. Discussion

In nature, more than 80% of ambient temperatures are below 5°C, while in the north and south poles, the average sea temperature is −1.8°C, and even in summer, the average soil temperature does not exceed 10°C. A large variety of microorganisms exist at such low temperatures, of which bacteria are the most diverse. Cold-adapted, also know as psychrophilic, bacteria have adapted to these cold environments. These bacteria have unique metabolic mechanisms, controlled by unique enzymes. Cold-adapted bacteria have gained the ability to produce cold-adapted enzymes during long-term evolutionary processes. Cold-adapted enzymes have high molecular structural flexibilities and low activation energies and, therefore, can catalyze enzymatic reactions at high rates at low temperatures. Consequently these enzymes have a wide range of applications in various fields.

More than 100 cold-adapted enzymes have been successfully expressed so far, and these cold-adapted enzymes have very good application prospects in food processing, the detergents industry, medical field, environmental protection, and basic molecular biology research. But most cold-adapted enzymes have been found from cultured microorganisms or from the metagenomes of uncultured microorganisms and are primarily derived from deep-sea oceans, high-elevation plains and mountain soils, or polar region habitats. Owing to these extreme environments, many of these psychrophilic microorganisms are difficult to culture, which makes the isolation, purification, and catalytic mechanism research of cold-adapted enzymes from these microorganisms particularly difficult. In addition, presently the unsolved problem remains that most cold-adapted enzymes are quite unstable in structure making it difficult to fully realize industrial production and application. The amino acid sequence, secondary structure, and the higher structure of cold-adapted enzymes are different from those of mesophilic and thermophilic enzymes. It is these structural characteristics that lead to a looser structure, less stability, and higher modification requirements of cold-adapted enzymes.

Enzyme immobilization and chemical and physical modification methods to address these issues affect the catalytic efficacy of the enzymes to a certain extent. Most modifications of cold-adapted enzymes are based on molecular biology and use rational or irrational design protocols. Modification methods for mesophilic and thermophilic enzymes have often been reported using these methods. However, cold-adapted enzyme modification by saturation mutagenesis and fixed-point mutation based on computer prediction and irrational design are less reported. This severely limits the development of cold-adapted enzyme modification and application. Therefore, we discuss cold catalysis mechanisms and strategies for improving the stability of cold enzymes in this review, hoping to lay a theoretical foundation for the research and application of cold-adapted enzymes in the future.

## Author contributions

YL, NZ, QZ, and SZ: conceptualization. YL, NZ, JM, QW, QZ, and SZ: visualization. YL: writing-original draft. YL, SZ, CT, YF, GC, and RZ: writing-review and editing. All authors contributed to the article and approved the submitted version.

## Funding

This work was supported by the Natural Science Foundation of Science and Technology Department of Jilin Province (grant number: 20220101334JC), the Chinese Academy of Sciences Strategic Pilot Science and Technology Project XDA28020400, the Key Projects of the Jilin Province Science and Technology Development Plan (grant number: 20210203117SF).

## Conflict of interest

The authors declare that the research was conducted in the absence of any commercial or financial relationships that could be construed as a potential conflict of interest.

## Publisher’s note

All claims expressed in this article are solely those of the authors and do not necessarily represent those of their affiliated organizations, or those of the publisher, the editors and the reviewers. Any product that may be evaluated in this article, or claim that may be made by its manufacturer, is not guaranteed or endorsed by the publisher.
